# Intraoperative Bacterial Contamination and Activity of Different Antimicrobial Prophylaxis Regimens in Primary Knee and Hip Replacement

**DOI:** 10.3390/antibiotics10010018

**Published:** 2020-12-27

**Authors:** Alba Rivera, Alba Sánchez, Sonia Luque, Isabel Mur, Lluís Puig, Xavier Crusi, José Carlos González, Luisa Sorlí, Aránzazu González, Juan Pablo Horcajada, Ferran Navarro, Natividad Benito

**Affiliations:** 1 Department of Microbiology, Hospital de la Santa Creu i Sant Pau—Institut d’Investigació Biomèdica Sant Pau, 08025 Barcelona, Spain; mrivera@santpau.cat (A.R.); asanchezmor@santpau.cat (A.S.); FNavarror@santpau.cat (F.N.); 2 Department of Genetic and Microbiology, Universitat Autònoma de Barcelona, 08193 Barcelona, Spain; 3 Department of Pharmacy, Hospital del Mar—Hospital del Mar Medical Research Institute (IMIM), 08003 Barcelona, Spain; sluque@parcdesalutmar.cat; 4 Department of Medicine, Faculty of Medicine, Universitat Autònoma de Barcelona, 08193 Barcelona, Spain; imur@santpau.cat (I.M.); jhorcajada@parcdesalutmar.cat (J.P.H.); 5 Infectious Disease Unit, Department of Internal Medicine, Hospital de la Santa Creu i Sant Pau—Institut d’Investigació Biomèdica Sant Pau, 08025 Barcelona, Spain; 6 Bone and Joint Infection Study Group of the Spanish Society of Infectious Diseases and Clinical Microbiology (GEIO-SEIMC), 28003 Madrid, Spain; lsorli@parcdesalutmar.cat; 7 Department of Orthopedic Surgery and Traumatology, Hospital del Mar—Hospital del Mar Medical Research Institute (IMIM), 08003 Barcelona, Spain; lpuig@parcdesalutmar.cat; 8 Department of Orthopedic Surgery and Traumatology, Hospital de la Santa Creu i Sant Pau—Institut d’Investigació Biomèdica Sant Pau, 08025 Barcelona, Spain; XCrusi@santpau.cat (X.C.); JGonzalezR@santpau.cat (J.C.G.); agonzalezo@santpau.cat (A.G.); 9 Department of Infectious Diseases, Hospital del Mar—Hospital del Mar Medical Research Institute (IMIM), 08003 Barcelona, Spain; 10 Department of Experimental and Health Sciences, Universitat Pompeu Fabra, 08003 Barcelona, Spain

**Keywords:** surgical antimicrobial prophylaxis, knee arthroplasty, hip arthroplasty, prosthetic joint infection, surgical site infection prevention, prosthetic joint infection prevention, intraoperative cultures, antibiotic levels, serum bactericidal titer

## Abstract

Surgical antimicrobial prophylaxis (SAP) is important for the prevention of prosthetic joint infections (PJIs) and must be effective against the microorganisms most likely to contaminate the surgical site. Our aim was to compare different SAP regimens (cefazolin, cefuroxime, or vancomycin, alone or combined with gentamicin) in patients undergoing total knee (TKA) and hip (THA) arthroplasty. In this preclinical exploratory analysis, we analyzed the results of intraoperative sample cultures, the ratio of plasma antibiotic levels to the minimum inhibitory concentrations (MICs) for bacteria isolated at the surgical wound and ATCC strains, and serum bactericidal titers (SBT) against the same microorganisms. A total of 132 surgical procedures (68 TKA, 64 THA) in 128 patients were included. Cultures were positive in 57 (43.2%) procedures (mostly for coagulase-negative staphylococci and *Cutibacterium* spp.); the rate was lower in the group of patients receiving combination SAP (adjusted OR 0.475, CI95% 0.229–0.987). The SAP regimens evaluated achieved plasma levels above the MICs in almost all of intraoperative isolates (93/94, 98.9%) and showed bactericidal activity against all of them (SBT range 1:8–1:1024), although SBTs were higher in patients receiving cefazolin and gentamicin-containing regimens. The potential clinical relevance of these findings in the prevention of PJIs remains to be determined.

## 1. Introduction

Prosthetic joint infection (PJI) is a serious complication associated with substantial morbimortality and economic costs [1]. Microorganisms introduced at the time of surgery, contiguous spread from adjacent infected tissue, and hematogenous seeding from a remote site are considered the usual routes of infection, although the former is believed to be the most frequent [1]. The risk of infection developing after microbial contamination of the surgical field depends on the dose and virulence of the pathogen and the patient’s resistance to infection [2]. Surgical antimicrobial prophylaxis (SAP), considered to be one of the most important preventive strategies, can help offset this by reducing the risk of surgical site infections (SSIs), including PJIs [3,4]. The goal of SAP is to eradicate bacteria inoculated into the wound at the time of surgery. From a pharmacodynamic point of view, antimicrobial levels should be maintained above the minimal inhibitory concentration (MIC) of the pathogens most likely to contaminate the surgical field for the whole duration of the operation [5,6,7]. Cefazolin or cefuroxime (first- and second-generation cephalosporins, respectively) and vancomycin in cases of beta-lactam allergy, are the antibiotics most commonly used and recommended in current guidelines, although there are no data supporting the superiority of one class of antimicrobials over another for SAP in total joint replacement [5,6]. Furthermore, studies have suggested that a growing proportion of SSIs (including PJIs) following arthroplasty procedures are caused by organisms resistant to first- and second-generation cephalosporins, including both Gram-positive (mainly methicillin-resistant staphylococci), and Gram-negative bacteria (such as some Enterobacterales or *Pseudomonas aeruginosa*) [8,9,10,11]. In light of this, various expanded combination SAP regimens have been proposed and analyzed in small clinical studies, with different effects but no conclusive results because of their methodological limitations [12,13,14,15,16,17]. Consequently, routine prophylactic use of dual antibiotics (such as cephalosporins and aminoglycosides or cephalosporins and vancomycin) is not currently recommended [18].

Conclusively demonstrating the superiority of one SAP regimen over another in clinical studies involves overcoming a number of problems. Ideally, randomized controlled trials would be conducted, but these would require an extraordinarily large number of participants (thousands) due to the relatively low incidence of PJI (1–2%). Furthermore, follow-up duration would be extremely long—at least two years—to take account of delayed cases of PJI. [19]. Before considering any clinical trial, therefore, the prophylactic regimens to be compared should be carefully evaluated. A preclinical exploratory analysis of potential SAP regimens using microbiological, pharmacokinetic (PK), and pharmacodynamic (PD) studies could be a very useful step. Using this approach, the aim of our study was to compare intraoperative bacterial contamination and the activity of six SAP regimens against microorganisms isolated in the surgical wounds of patients undergoing elective primary total knee (TKA) and hip (THA) arthroplasty surgery. We analyzed the following data obtained at the end of surgical procedures: (1) bacteria isolated from surgical wounds (rate and etiology); (2) free plasma antibiotic concentrations relative to the MICs of the isolated microorganisms and some reference American Type Culture Collection (ATCC) strains; and (3) serum bactericidal titers (SBTs) against the same microorganisms.

## 2. Patients and Methods

### 2.1. Setting and Patients

This prospective study was conducted at two acute care university hospitals in Barcelona, Spain (Hospital de la Santa Creu i Sant Pau and Hospital del Mar). The Institutional Review Boards of the two participating hospitals approved the study.

Patients undergoing elective primary total knee and hip replacement surgery between June 2016 and March 2020 were included. Three orthopedists recruited patients who agreed to participate in the study and provided written informed consent. Each of the four cephalosporin-containing regimens was sequentially administered to consecutively enrolled patients; penicillin-allergic patients received vancomycin or vancomycin and gentamicin. Preoperative whole-body bathing or showering with chlorhexidine soap on the day of the surgical procedure and the night before was indicated. Alcoholic 2% chlorhexidine was used as antiseptic for skin preparation before surgical incision.

A minimum follow-up of one year was planned after prosthesis implantation in order to diagnose possible postoperative PJIs; this minimum period of follow-up is still ongoing in some patients.

### 2.2. Surgical Antimicrobial Prophylaxis Regimens

Patients received cefazolin (2 g), cefuroxime (1.5 g), or vancomycin (15 mg/kg total body weight), alone or in combination with gentamicin (5 mg/kg total body weight) as SAP. Antibiotics were administered intravenously within 60 min prior to incision, except for vancomycin, which was given up to 120 min prior to incision.

### 2.3. Sample Collection

Blood samples (3–5 mL) were collected at the end of surgery in heparinized and gelose-containing tubes. After centrifugation, serum and plasma samples were stored at −80 °C ± 5 °C until testing for antimicrobial levels and SBT titers.

Five standard perioperative tissue samples were collected from each patient at the end of surgery and sent for culture. All samples were obtained after implantation of the prosthesis and before wound closure. In TKA surgery, two tissue samples were collected from around the femur, two from around the tibia, and one from the subcutaneous tissue. In THA surgery, two tissue samples were collected from around the acetabulum, two from around the femur, and one from the subcutaneous tissue.

### 2.4. Determination of Antibiotic Levels

Plasma concentrations of cefazolin and cefuroxime were determined by a validated high-performance liquid chromatography (HPLC) method with a UV-Vis spectrophotometric detector, and those of gentamicin and vancomycin by chemiluminescent microparticle immunoassay (Alinity, Abbott). For the HPLC assay, 100 µL of each plasma sample was mixed with 200 µL of methanol and vortexed for 10 s. The mixture was then centrifuged for 5 min at 15,000× *g* in a refrigerated centrifuge and 20 µL of the supernatant was injected into the system for the assay (Alliance e2695, and 2487 HPLC Absorbance UV-Vis Detector, Waters). The method was shown to be sensitive and specific for the measurement of cefazolin and cefuroxime in plasma. The assay response was linear (coefficient of linearity >0.99) over the full range of concentrations assayed (0.5–200 mg/L for cefazolin and 0.5–100 mg/L for cefuroxime). The limit of quantification was 0.5 mg/L for both cefazolin and cefuroxime. Imprecision values were < 15% over the entire range of calibration standards, and accuracy was within the range of 85–115% for all concentrations. Total measured concentrations of cefazolin, cefuroxime and vancomycin were adjusted to free concentrations, assuming protein binding of 80%, 40% and 50%, respectively [20,21]. Protein binding of gentamicin was considered to be negligible [21,22].

Antibiotic levels were considered appropriate when their free plasma concentration was above the MIC of pathogens isolated from the wound at the time of the prosthetic joint implant surgery, or the MIC of the ATCC strains studied.

### 2.5. Microbiological Methods

Tissue samples were homogenized in 1 ml of sterile saline using a sterile mortar and pestle, and 100 µl volumes were inoculated onto each plate of blood agar (BioMerieux, Marcy l’Etoile, France) and chocolate agar (BioMerieux, Marcy l’Etoile, France), both incubated in aerobic conditions, and Schaedler agar (BioMerieux, Marcy l’Etoile, France) incubated in anaerobic conditions. The remaining homogenate was inoculated into thioglycollate broth. Cultures were incubated for seven days at 35 ± 2 °C. Bacterial isolates were identified using MALDI-TOF (Bruker, Bremen, Germany). Antimicrobial susceptibility was determined by either gradient diffusion (Liofilchem, Roseto degli Abruzzi, Italy) or disk diffusion (Rosco Diagnostica, Taastrup, Denmark) and interpreted according to EUCAST [23]. Bacterial isolates were tested against the antibiotics used in each prophylaxis. For staphylococci, resistance to cefazolin or cefuroxime was inferred from resistance to cefoxitin.

While microbiological diagnosis of PJI requires that at least two of a minimum of five intraoperative cultures (obtained at the surgery to treat the infection) yield the same microorganism, however the present study represented a different scenario. Prosthetic joint implantation is clean surgery, and therefore, a very low bacterial inoculum is expected in the surgical field. For this reason, we considered any growth on any of the plates as a positive culture, and a patient with a single positive culture was rated as having a positive intraoperative culture. Culture-positive results were blinded, and patients were not given antimicrobial treatment on the basis of these results. The only antibiotic administered to patients was the surgical prophylaxis.

SBT was performed with sera collected at the time of surgical closure from patients with positive intraoperative cultures and measured against the patient’s respective bacterial isolates. In addition, SBT was performed with sera from patients with positive intraoperative cultures and a subset of patients with negative cultures against the reference strains *Staphylococcus epidermidis* ATCC 12228, *Staphylococcus aureus* ATCC 25923, *Escherichia coli* ATCC 25922 and *P. aeruginosa* ATCC 27853. The assays were performed by the microdilution method, according to the Clinical Laboratory Standards Institute guidelines [24], with some modifications.

Two-fold serial dilutions of patient serum were prepared in cation-adjusted Mueller Hinton broth (Thermo Scientific, USA) or Mueller Hinton supplemented with lysed horse blood (Thermo Scientific, USA). The dilution range was 1:2–1:1024. Plates were incubated at 35 ± 2 °C for 24 h or 48 h. The SBT titer was defined as the highest dilution of patient serum at which a ≥99.9% reduction in the starting inoculum was achieved. Reciprocal SBT values were used to calculate median SBTs.

### 2.6. Statistical Methods

Categorical variables were summarized as percentages of the total sample for that variable, and continuous variables as means and standard deviation (SD) or median and interquartile range (IQR), depending on their homogeneity. The Wilcoxon rank-sum and Chi-squared tests (or Fisher’s exact tests when appropriate) were used to evaluate group differences for continuous and categorical variables, respectively. A multivariate logistic regression model was used to identify factors independently associated with a higher risk of having positive intraoperative cultures. Any variable tested in univariate analysis with a *p*-value less than 0.25, together with all variables of known clinical importance, were selected as candidates for the first multivariate model. We then followed the purposeful selection of covariates method described by Hosmer and Lemeshow [25]. Final parameter estimates are shown as odds ratios (ORs) with their corresponding 95% confidence intervals (CIs). *p*-values of < 0.05 were considered to be significant for all statistical tests. Data were analyzed using IBM^®^ SPSS^®^, version 26.0.

## 3. Results

### 3.1. Patients and Surgical Antimicrobial Prophylaxis

A total of 132 surgical procedures for joint replacement (68 TKA and 64 THA) were performed in 128 patients (four patients underwent two different procedures at different times). Seventy-two (56.3%) patients were female, and the median age was 71 years (SD 8.6) (Table 1). The SAP regimens administered were: cefazolin, in 22 (16.7%) procedures, cefuroxime in 20 (15.2%), vancomycin in 11 (8.3%), cefazolin plus gentamicin in 39 (29.5%), cefuroxime plus gentamicin in 20 (15.2%) and vancomycin plus gentamicin in 20 (15.2%).

During a median follow-up of 15 months (interquartile range, IQR, of 21), two PJIs (1.5%) were diagnosed. A 72-year-old woman with no underlying pathology, BMI 33, ASA II, and an uneventful 88-min surgery in which she received cefuroxime as prophylaxis, presented a THA infection caused by *S. aureus* (methicillin-susceptible) five weeks after prosthesis implantation. Free plasma concentration of cefuroxime at the end of the surgery was 9 mg/L. The second was a TKA infection caused *by Morganella morganii*, which occurred one month after a 100-min surgery. The patient was a 74-year-old diabetic woman, BMI 38.5 and ASA III, who received cefazolin plus gentamicin as SAP. In this case, free plasma concentration of cefazolin was 15.4 mg/L and gentamicin 15.2 mg/L. Both patients had negative intraoperative cultures during prosthesis implantation.

### 3.2. Intraoperative Cultures

At least one of the five tissue samples taken yielded positive culture results in 57 (43.2%) surgical procedures: 39.7% (27/68) were TKA and 46.9% (30/64) THA. The number of positive samples per patient ranged from one to five (median 2, IQR 1). There were no substantial differences in culture yield between subcutaneous tissue samples (20 positive culture samples from 57 procedures, 35.1%) and those from deep tissue (the four deep samples yielded positive cultures in 25, 17, 20 and 25 cases, respectively, with a mean of 21.8, 38.2%).

Table 1 shows the characteristics of patients undergoing primary THA and TKA, with and without positive intraoperative cultures. With respect to single-drug prophylaxis, patients receiving cefazolin had the lowest percentage of positive cultures, while patients with combined SAP regimens less frequently had positive intraoperative cultures than those with a single drug, although these differences were not statistically significant. In the adjusted analysis, we found that males had a two-fold higher risk of positive cultures than women, while gentamicin-containing SAP regimens were associated with a lower risk of positive cultures.

Overall, a total of 94 bacterial isolates—all of them Gram-positive bacteria—were identified. The most frequently isolated microorganisms were coagulase-negative staphylococci (CoNS), 42 (44.7%), followed by *Cutibacterium* spp., 34 (36.2%). The predominant individual species was *Cutibacterium acnes* (35.1%). Polymicrobial isolation occurred in 23 (40.4%) culture-positive surgical procedures (14 of 30 THA [46.7%] and 9 of 27 TKA [36.3%]; *p* = 0.451). *Cutibacterium* spp. or CoNS were isolated in more than half of culture-positive surgeries (Table 2). *Cutibacterium* spp. was more frequently found in THA than in TKA surgery.

### 3.3. Susceptibility of Bacterial Isolates and ATCC Strains, Antibiotic Plasma Levels and Serum Bactericidal Titers

Supplementary Table S1 shows in detail the following data of patients with intraoperative positive cultures: plasma levels of antibiotics used as SAP, bacteria isolated and the corresponding MICs of the antimicrobials administered, and SBT against the isolated bacteria.

Cefazolin MICs determined in 38 bacterial isolates obtained from patients receiving this antibiotic (with or without gentamicin) ranged from 0.032–64 mg/L. There were five (13.2%) cefazolin-resistant isolates, of which four were CoNS and one was *Paenibacillus lautus*. Cefuroxime MICs for 37 isolates ranged from 0.016 to 16 mg/L, one (2.6%) of which was resistant (*S. epidermidis*). MICs of vancomycin were determined in 16 isolates with a range of 0.125–2 mg/L; none of the isolates showed resistance. MICs of gentamicin for 42 strains ranged from 0.047 to 24 mg/L, with 22 (52.4%) resistant isolates (*C. acnes* and one *Staphylococcus warneri*).

Overall, 94.5% (86/91) of bacterial isolates were susceptible to the particular SAP regimen administered (or to at least one of the antibiotics in a combination regimen). With respect to single-drug cephalosporin prophylaxis, 82.3% (14/17) and 96% (24/25) of isolates were susceptible to cefazolin and cefuroxime, respectively. The rate of susceptible isolates was higher for combinations with cephalosporins plus gentamicin: 95.2% (20/21) in the case of cefazolin, and 100% (13/13) in the case of cefuroxime, although these differences were not statistically significant. Plasma levels of antimicrobials used in prophylaxis were determined in 130 (98.5%) patients (blood samples could not be obtained from two patients). Median plasma levels and ratios to MIC are shown in Table 3.

Free plasma concentrations of cefazolin exceeded the MIC in 94.7% (36/38) of the isolates tested. Only two isolates (*P. lautus* and *S. warneri*) presented MICs above the plasma concentration. In the case of cefuroxime and vancomycin, free plasma concentrations were higher than the MICs in all isolates tested. Gentamicin plasma levels were higher than the MIC in all isolates except eight (seven strains of *C. acnes* and one strain of *S. warneri*), 80.9% (34/42). In all these cases, except for *S. warneri*, the plasma concentrations of antibiotic used in combination with gentamicin were above the MIC.

**Figure 1:**
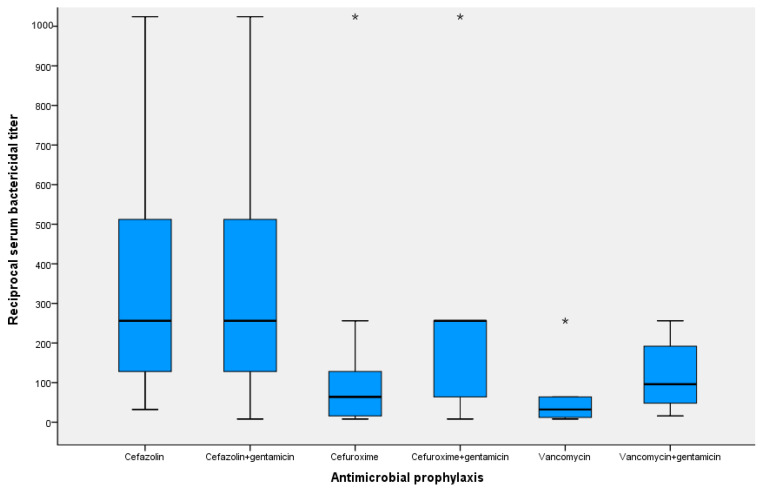
Reciprocal serum bactericidal titers against bacteria isolated in the surgical field for each surgical antimicrobial prophylaxis regimen. * Outliers are marked with an asterisk (*); outlier is defined as a data point that is located outside 1.5 times the interquartile range above the upper quartile and bellow the lower quartile.

SBTs were performed with serum samples obtained from patients with positive intraoperative cultures against the bacteria isolated from the surgical field of each patient (Figure 1, Table 4, and Supplementary Table S1). In four patients, SBT could not be performed due to a lack of serum.

Overall, SBTs ranged from 1:8 to 1:1024. Statistically significant differences between the six SAP regimens studied (*p* < 0.001) were observed. Among patients receiving single-drug prophylaxis, SBTs were higher with cefazolin than with both cefuroxime and vancomycin (*p* = 0.001 and *p* = 0.002, respectively), while no differences were observed between cefuroxime and vancomycin (*p* = 0.278). Globally, patients receiving combined prophylaxis with gentamicin had higher SBTs than those receiving single-drug prophylaxis (*p* = 0.009), although these differences were only relevant with cefuroxime (vs. cefuroxime plus gentamicin) (*p* = 0.023) and vancomycin (vs. vancomycin plus gentamicin) (*p* = 0.098), and were not observed with cefazolin (vs. cefazolin plus gentamicin) (*p* = 0.780). Of note, serum bactericidal activity was detected (SBTs ranging from 1:16 to 1:128) in four methicillin-resistant CoNS isolates from patients who received only cefazolin or cefuroxime (despite the fact that methicillin resistance implies resistance to all beta-lactams, cephalosporins included). Moreover, an SBT of 1:16 was found against one *S. warneri* isolate, which was the only one in which plasma levels of both prophylactic antibiotics (cefazolin and gentamicin) did not exceed the MIC (Supplementary Table S1).

The bactericidal activity of each SAP regimen was also assessed by comparing SBTs performed against the reference strains *S. epidermidis* ATCC 12228, *S. aureus* ATCC 25923, *E. coli* ATCC 25922 and *P. aeruginosa* ATCC 27853 (Table 4). For this, 93 sera samples (53 from patients with positive intraoperative cultures and 40 with negative cultures) were tested. The results of SBTs against the Gram-positive bacteria *S. epidermidis* and *S. aureus* were very similar to those observed against isolates taken from the surgical field (all of them also Gram-positive bacteria). Overall, patients receiving gentamicin-containing SAP regimens had higher SBT titers than those who received single-agent prophylaxis, although this difference was not observed in the cefazolin groups. With respect to single-drug prophylaxis, the highest SBTs were found for cefazolin. Bactericidal activity against the Gram-negative bacterium, *E. coli* ATCC 25922, was observed with all SAP regimens, except for vancomycin alone (because of the intrinsic resistance to vancomycin of Gram-negative bacteria). SBTs against this *E. coli* strain followed the same pattern as for Gram-positive bacteria (highest SBT titers with cefazolin groups, and higher SBTs with gentamicin-containing cefuroxime and vancomycin regimens than with single cefuroxime and vancomycin prophylaxis); however, all SAP regimens (except vancomycin alone) showed four-fold lower median titers than against Gram-positive bacteria. Bactericidal activity against the Gram-negative bacterium *P. aeruginosa* ATCC 27853 was only observed in sera from patients treated with combinations with gentamicin (which correlates with the intrinsic resistance of this strain against cefazolin, cefuroxime and vancomycin), but with median SBTs four- to eight-fold lower than against *E. coli* ATCC 25922.

Antibiotic plasma levels and MICs of drugs used in prophylaxis against the reference strains are shown in Table 5. For *P. aeruginosa* ATCC 27853, none of the antibiotics except gentamicin achieved plasma levels above the MIC. For the remaining reference strains tested, all the antibiotics showed plasma levels above the MIC, except for vancomycin and *E. coli* ATCC 25922.

## 4. Discussion

Antimicrobial prophylaxis plays a crucial role in reducing the incidence of PJIs, although there is no consensus about antibiotic choice [26]. Some observational clinical studies have analyzed the effect of different SAP regimens on SSI/PJI rates following arthroplasty surgery, with conflicting results. Babu et al. compared five different antimicrobial prophylactic regimes in elective primary TKA and found no differences in the incidence of PJI or the pathogens involved [27]. Wyles et al. evaluated different SAPs in patients undergoing primary TKA or THA and found higher rates of PJI when non-cefazolin antibiotics were used [28]. Tornero et al. found a significant decrease in the PJI rate when teicoplanin was added to cefuroxime during primary arthroplasty, thanks to the decrease in Gram-positive bacterial infections [13]. Similar results were reported by Barbero-Allende et al. with the addition of teicoplanin to cefazolin [17]. Another study found that the addition of gentamicin to cefazolin (or vancomycin in penicillin-allergic patients) reduced the SSI rate following THA [15]. These studies, however, have significant methodological limitations that prevent definitive conclusions from being drawn. Due to the difficulty of conducting sound clinical trials to compare the effect of different SAPs on PJI prevention, we evaluated six prophylactic regimens (cefazolin, cefuroxime and vancomycin as single agents or combined with gentamicin) in a preclinical exploratory study using microbiological and PK/PD analysis. We compared contamination of the surgical field, plasma antibiotic levels relative to the MICs of microorganisms isolated in wounds and some reference ATCC strains, and SBTs against the same bacteria.

Despite advances in preventive measures, intraoperative contamination of the surgical field in orthopedic surgery remains frequent. Contamination can originate from many sources, including the patients’ microbiota, surgical personnel, surgical instruments, or the operating room environment [29,30,31]. Our results showed an overall intraoperative contamination rate of 43.2%, consisting of Gram-positive bacteria often found in normal cutaneous microbiota. This percentage is in the upper range limit of rates observed in prior studies [32,33,34,35,36], although neither the number of samples per patient, nor the collection method or specific anatomical location were standardized and indeed varied widely between studies. Furthermore, fewer samples per patient were taken and the swab was the most frequent collection method, which has lower sensitivity and specificity than tissue samples [37]. In accordance with previous studies, the most frequent organisms isolated were CoNS and *C. acnes*, both of which form part of the skin microbiota and are considered to be of low virulence, although they are a common cause of PJI, especially CoNS [9,38]. After a median follow-up of 15 months, two patients (1.5%) developed PJI. In both cases, previous intraoperative cultures were negative. According to these results, and those observed in previous studies, intraoperative contamination during primary TKA and THA surgery is common, but cannot be used to identify patients at increased risk of PJI [32,33,34,35,36]. On the other hand, factors such as longer duration of surgery [35] and high body mass index [32] have been associated with an increased risk of contamination. Other studies have shown that the use of iodinated drapes reduced intraoperative contamination in patients undergoing primary knee arthroplasty [39]. In our study, after adjusting for clinically relevant variables, we found that the group of patients receiving gentamicin-containing SAP combinations had a lower percentage of positive intraoperative cultures than the group that received only one drug. Nevertheless, the potential clinical relevance of these results and their influence on the risk of developing PJI remain to be determined. In fact, because the influence of intraoperative contamination on SSIs has not been conclusively proven, one publication has posited a new hypothesis about the pathogenesis of SSI [40]. The authors proposed that pathogens located in areas remote from the SSI, such as the teeth or gastrointestinal tract, could be transported in immune cells (macrophages or neutrophils) to the wound site and cause wound infection. We agree with the authors that further studies using genetic approaches can help to more clearly determine the significance of intraoperative contamination or other potential sources of infection in order to improve the SSI prevention strategies.

We analyzed the possible usefulness of SBT to evaluate the activity of antimicrobial agents used in prophylaxis. SBT assesses the antibacterial activity of a drug in the patient’s serum [41,42]. These tests have been used in the past to guide antimicrobial therapy in severe infections such as endocarditis and osteomyelitis, but are practically abandoned in routine contemporary clinical practice because they are technically demanding and their usefulness has been questioned. Nevertheless, the advantage of SBT over standard antimicrobial susceptibility methods is that it integrates PK/PD factors. Indeed, some studies have breathed new life into this technique by showing its usefulness for monitoring antimicrobial therapy in patients with difficult-to-treat or multidrug-resistant infections [43,44,45]. Although SBT titers of 1:8 have been reported to correlate with successful outcomes of infection [41,42], the SBT titer required for surgical prophylaxis is unknown. Considering the breakpoint accepted for therapeutics, our study found that bactericidal activity was maintained throughout the surgical procedure against all isolates recovered from intraoperative samples (SBT range 1:8–1:1024), regardless of the prophylaxis used. Among the reference ATCC strains tested, staphylococci corroborated these results. For Gram-negative reference strains, bactericidal activity was observed against *E. coli* ATCC 25922 with all prophylactic regimens except vancomycin, while activity against *P. aeruginosa* ATCC 27853 was observed only with gentamicin combinations. These results correlate with the intrinsic resistance of both species to vancomycin, as well as the additional intrinsic resistance of *P. aeruginosa* to cefazolin and cefuroxime. The consistency of the results obtained using SBT supports its potential utility for assessing SAP.

Although high rates of resistance to beta-lactams have been found among pathogens causing PJI [8,10,38,46], particularly CoNS, most of the bacteria cultured from intraoperative samples in our study were susceptible to the cephalosporins administered. SAP may be able to eliminate these susceptible strains, but may also select for resistant ones that could cause PJI. Interestingly, the SBTs in patients receiving cefazolin or cefuroxime alone were particularly high against methicillin-resistant staphylococci. This could be related to our finding that antibiotic plasma levels at the end of the surgical procedure were well above the MICs for the organisms encountered in intraoperative cultures, which is considered to be the goal of SAP [5,6]. This, in conjunction with the low bacterial load, would be enough to achieve bacterial eradication. Nevertheless, bactericidal activity against Gram-positive isolates was obtained even in cases where antimicrobial plasma concentrations did not exceed or were slightly above the MIC. This was also true for methicillin-resistant staphylococci isolates, which suggests that currently recommended prophylactic regimens with cefazolin or cefuroxime continue to show activity even against these resistant Gram-positive bacteria. However, as expected, bactericidal activity was not enough against some Gram-negative isolates such as *P. aeruginosa*—intrinsically resistant to first- and second-generation cephalosporins and vancomycin—showing high MICs that greatly exceed the plasma concentration. Combination prophylaxis with gentamicin could play a role against these microorganisms or other cefazolin- or cefuroxime-resistant Gram-negative bacteria. This could be particularly relevant because some studies have reported an increased frequency of Gram-negative bacilli causing PJIs [8]. Furthermore, we found that the addition of gentamicin increased the antimicrobial activity of cefuroxime and vancomycin against bacteria isolated from surgical wounds, as well as ATCC staphylococci and *E. coli* reference strains. Cefazolin had higher activity than cefuroxime or vancomycin. Although the potential clinical implications of these findings need to be clarified, they should be borne in mind in order to design additional studies about arthroplasty surgery prophylaxis.

This study has some limitations. In the analysis of intraoperative cultures, any number of colonies was considered positive, which may have led to overestimating the positive culture rate in the surgical field. Bacterial contamination can occur at any time during analytical sample processing, and this possibility cannot be ruled out. Conversely, the lack of bacterial growth does not necessarily imply surgical site sterility because of the limitations of current techniques in detecting all bacteria present in the surgical field. We did not randomly assign patients to receive the different SAP regimens. While randomization is expected to produce comparable intervention groups and eliminate potential sources of bias in treatment assignment, this cannot be excluded in the present study. To overcome this limitation, we adjusted for clinically relevant covariates in the analysis stage; however, we cannot rule out the potential effect of unknown confounding or prognostic variables. Furthermore, although we performed an extensive microbiological and PK/PD study with different SAPs and found consistent results, its applicability in the prevention of SSIs/PJIs remains to be determined. It should also be considered that SAP is only part of the measures for prevention of SSI and that a patient’s intrinsic characteristics and perioperative factors have a major influence on the development of these infections.

In conclusion, the six antimicrobial prophylactic regimens evaluated (cefazolin, cefuroxime and vancomycin, alone and combined with gentamicin) showed good activity against the microorganisms isolated from intraoperative tissue samples—including cephalosporins against methicillin-resistant CoNS—and achieved plasma levels above the MICs in almost all of them. Intraoperative bacterial contamination was less frequent in the combination group than in the group receiving single-drug prophylaxis. Although all the prophylactic regimens showed good activity against the intraoperative bacteria and staphylococcal reference strains (all of them Gram-positive bacteria), cefazolin with or without gentamicin displayed the greatest activity; cefuroxime and vancomycin as single drugs had lower activity than when combined with gentamicin. With respect to Gram-negative bacteria, SBT demonstrated, as expected, that vancomycin alone was the only SAP without activity against the *E. coli* reference strain, and that only gentamicin-containing regimens were active against the *P. aeruginosa* reference strain. The potential clinical relevance of these findings in the prevention of PJI remains to be determined. SBT was shown to be a potentially reliable tool for assessing antimicrobial surgical prophylaxis.

## Figures and Tables

**Table 1:** Patients undergoing primary total knee and hip arthroplasty surgical procedures, with and without positive intraoperative cultures.

Variable	Intraoperative Cultures		*p*-Value	Multivariate Analysis	*p*-Value
	Positive (*n* = 57)	Negative (*n* = 75)		OR (CI 95%)	
Sex—number of males or females with positive cultures/total number of males or females, respectively (%)			0.023	**2.412 (1.170–4.973)**	**0.017**
- Male	31/57 (54.4)				
- Female	26/75 (37.7)				
Age, years—mean (SD)	71 (9.6)	72 (7.9)	0.615		
BMI—mean (SD)	29.9 (5.1)	29.1 (4.9)	0.393		
Antimicrobial prophylaxis—number of culture-positive patients with each type of prophylaxis/total of patients receiving each type of prophylaxis (%)					
- Cefazolin - Cefuroxime - Vancomycin	9/22 (40.9) 13/20 (65) 6/11 (54.5)		0.293		
- Cefazolin - Cefazolin + gentamicin	9/22 (40.9) 14/39 (35.9)		0.698		
- Cefuroxime - Cefuroxime + gentamicin	13/20 (65) 9/20 (45)		0.204		
- Vancomycin - Vancomycin + gentamicin	6/11 (54.5) 6/20 (30)		0.180		
- Cefazolin, cefuroxime, and vancomycin - Cefazolin + gentamicin, cefuroxime + gentamicin and vancomycin + gentamicin	29/79 (36.7) 28/53 (52.8)		0.067	**0.475 (0.229–0.987)**	**0.046**
**Prosthesis location**—number of patients with a hip or knee prosthesis and positive cultures/total number of patients with hip or knee prostheses, respectively (%) - Hip - Knee	30/64 (46.9) 27/68 (39.7)		0.406		
**Surgery duration**, minutes—mean (SD)	75 (18.8)	78 (20.2)	0.363		

CI, confidence interval; OR, odds ratio.

**Table 2:** Bacterial species isolated from intraoperative samples during total hip and knee replacement surgical procedures with positive cultures.

Bacterial Species	Surgical Procedures (*n* = 57)	THA (*n* = 30)	TKA (*n* = 27)	*p*-Value *
*Cutibacterium* species—*n* (%)	34 (59.6)	22 (73.3)	12 (44.4)	**0.026**
- *Cutibacterium acnes*	33	21	12	0.051
- *Cutibacterium avidum*	1	1	0	
Coagulase-negative staphylococci—*n* (%)	30 (52.6)	15 (50)	15 (55.6)	0.675
- *Staphylococcus epidermidis*	19 (33.3)	8 (26.7)	11 (40.7)	0.399
- *Staphylococcus hominis*	12 (21.1)	8 (26.7)	4 (14.8)	0.441
- *Staphylococcus warneri* - *Staphylococcus simulans* - *Staphylococcus capitis* - *Staphylococcus caprae* - *Staphylococcus haemolyticus* - *Staphylococcus pettenkoferi* - *Staphylococcus saccharolyticus*	3 2 1 1 1 1 1	1 2 1 1 0 0 1	2 0 0 0 1 1 0	
*Micrococcus luteus*—n (%)	8 (14.0)	4 (13.3)	4 (14.8)	
*Corynebacterium* species—n (%)	4 (7.0)	3	1	
- *Corynebacterium afermentans* - *Corynebacterium pseudodiphteriticum* - *Corynebacterium accolens* - *Corynebacterium mucifaciens* - *Corynebacterium propinquum* - *Corynebacterium simulans*	1 1 1 1 1 1	0 1 1 1 1 0	1 0 0 0 0 1	
*Paenibacillus lautus*	1	1	0	
*Actinomyces neuii*	1	1	0	
*Dermabacter hominis*	1	0	1	
*Kocuria rhizophila*	1	1	0	

THA, total hip arthroplasty; TKA, total knee arthroplasty. * Statistically significant differences between percentages were considered when an organism or group of organisms was isolated in more than ten surgical procedures.

**Table 3:** Prophylactic plasma antimicrobial levels in culture-positive surgical procedures and ratios of these antimicrobial levels to the minimum inhibitory concentrations (MICs) for bacteria isolated in the surgical field.

Antimicrobial Used as Prophylaxis	Free Plasma Concentration (mg/L), Median (Range)	Free Plasma Concentration (mg/L)/ MIC (mg/L), Median (Range)
Cefazolin	17.3 (11.2–33.2)	44.4 (0.3–1037.5)
Cefuroxime	24.2 (11–44.2)	81.6 (1.1–1833.5)
Gentamicin	12.3 (8.5–19.4)	9.01(0.6–323.4)
Vancomycin	7.8 (4.6–19.05)	25.6(3.5–152.4)

**Table 4:** Reciprocal serum bactericidal titers against bacteria isolated in the surgical field and reference strains for each antimicrobial prophylaxis.

Antimicrobial Prophylaxis	Reciprocal Serum Bactericidal Titer—Median (Range)				
	Isolates from the Surgical Field	*Staphylococcus epidermidis* ATCC 12228	*Staphylococcus aureus* ATCC 25923	*Escherichia coli* ATCC 25922	*Pseudomonas aeruginosa* ATCC 27853
Cefazolin	256 (32–1024)	256 (32–512)	256 (64–1024)	64 (16–256)	<2 (<2)
Cefazolin+Gentamicin	256 (8–1024)	512 (32–1024)	256 (32–1024)	64 (16–256)	8 (<2–16)
Cefuroxime	64 (8–1024)	64 (16–512)	32 (8–64)	8 (2–32)	<2 (<2)
Cefuroxime+Gentamicin	256 (8–1024)	256 (64–512)	128 (8–128)	32 (16–32)	4 (<2–4)
Vancomycin	32 (8–256)	12 (8–32)	12 (8–16)	<2 (<2–2)	<2 (<2)
Vancomycin+Gentamicin	64 (16–256)	256 (256–512)	128 (32–256)	32 (16–64)	4 (4–8)

**Table 5:** Antibiotic plasma levels in surgical procedures with positive (*n* = 53) and negative (*n* = 40) intraoperative cultures and MICs of antimicrobial agents used in prophylaxis against ATCC reference strains.

Antimicrobial (n)	Free Plasma Concentration (mg/L), Median (Range)	MIC (mg/L)			
		*Staphylococcus epidermidis* ATCC 12228	*Staphylococcus aureus* ATCC 25923	*Escherichia coli* ATCC 25922	*Pseudomonas aeruginosa* ATCC 27853
Cefazolin (56)	17.3 (6.5–35.4)	0.5	0.5	3	>256
Cefuroxime (21)	25.7 (11–44.2)	0.75	0.5	6	>256
Gentamicin (54)	12.55 (8.5–19.4)	0.125	0.38	0.75	1.5
Vancomycin (16)	7.65 (4.6–19.05)	1.5	1	>256	>256

## Data Availability

The data presented in this study are available on request from the corresponding author.

## Supplementary Materials

The following are available online at https://www.mdpi.com/2079-6382/10/1/18/s1, Table S1: Antibiotic plasma levels of patients with intraoperative positive cultures, bacterial species from surgical samples, MICs of antimicrobials used in prophylaxis and serum bactericidal titers against the isolated bacteria.
