# Supplementary material for: Intraoperative Bacterial Contamination and Activity of Different Antimicrobial Prophylaxis Regimens in Primary Knee and Hip Replacement
Source: Antibiotics (Basel). 2020 Dec 27;10(1):18. doi: 10.3390/antibiotics10010018 (PMC7823842; doi:10.3390/antibiotics10010018)
Supplement: Supplementary file 1 [file antibiotics-10-00018-s001.pdf]

Supplementary Table 1. Antibiotic plasma levels of patients with intraoperative positive cultures, bacterial species from surgical samples, MICs of antimicrobials used in prophylaxis and serum bactericidal titers against the isolated bacteria.

Antimicrobial prophylaxis	Patient	Free plasma concentration (mg/L)				Bacterial species	MIC (mg/L)				Reciprocal SBT
		CFZ	GEN	CXM	VAN		CFZ	GEN	CXM	VAN	
Cefazolin	**02/004**	20.4				*Staphylococcus epidermidis*[1,2]	1				64
						Staphylococcus epidermidis[1]	0.38				256
						Corynebacterium afermentans	0.75				128
	**02/013**	19.9				*Staphylococcus epidermidis*[2]	2				128
	**02/014**	15.2				*Cutibacterium acnes*	0.5				256
	**02/016**	25.3				*Cutibacterium acnes*	0.125				ND
						Staphylococcus hominis[2]	3				ND
	**02/036**	15.2				*Staphylococcus epidermidis*	0.25				256
						Micrococcus luteus	0.38				256
	**02/038**	19.5				*Cutibacterium acnes*	0.25				512
	**02/040**	19.7				*Staphylococcus hominis*	0.38				512
						Cutibacterium acnes	0.25				64
	**02/046**	18.8				*Micrococcus luteus*	0.5				128
	**02/048**	33.2				*Staphylococcus epidermidis*	0.25				512
						Corynebacterium propinquum	0.032				1024
						Cutibacterium accolens	0.125				512
						Cutibacterium acnes	0.75				32
	**median (range)**	19.7 (15.2-33.2)					0.38 (0.032-3)				256 (32-1024)
Cefazolin + Gentamicin	**01/001**	15.8	12.2			*Cutibacterium acnes*	0.19	8			256
	**01/005**	17.4	**13.5**			*Cutibacterium acnes*	0.125	**24**			256
	**01/016**	17.3	12.1			*Staphylococcus hominis*	0.5	0.047			512

Antimicrobial prophylaxis	Patient	Free plasma concentration (mg/L)				Bacterial species	MIC (mg/L)				Reciprocal SBT
		CFZ	GEN	CXM	VAN		CFZ	GEN	CXM	VAN	
	**01/019**	16.6	13.7			*Staphylococcus epidermidis*	0.38	0.064			256
						Staphylococcus warneri[2]	**32**	**24**			16
	**01/021**	13.9	**11.5**			*Cutibacterium acnes*	0.19	**12**			128
	**01/023**	15.7	13			*Cutibacterium acnes*	0.19	8			512
	**01/027**	16.96	19.4			*Cutibacterium acnes*	0.38	8			128
	**01/032**	12.7	12.6			*Staphylococcus pettenkoferi*	2	0.064			1024
	**01/033**	18.8	14.2			*Staphylococcus hominis*	0.5	0.047			512
						Cutibacterium acnes	0.38	8			256
						Staphylococcus simulans	1.5	0.125			256
	**01/035**	16.8	**11.7**			*Cutibacterium acnes*	0.125	**12**			256
						Paenibacillus lautus	**64**	0.75			8
						Staphylococcus hominis	0.38	0.047			1024
	**01/036**	11.2	9.9			*Staphylococcus epidermidis*	0.5	0.094			256
						Corynebacterium pseudodiphteriticum	0.094	0.064			1024
						Cutibacterium acnes	0.19	8			128
						Staphylococcus hominis	0.5	0.047			32
	**01/048**	17.3	14			*Cutibacterium acnes*	0.25	8			128
	**01/040**	20.1	12.5			*Cutibacterium acnes*	0.5	8			512
	**01/024**	ND	ND			*Cutibacterium acnes*	ND	ND			ND
	**median (range)**	16.8 (11.2-20.1)	12.6 (9.9-19.4)				0.38 (0.094-64)	8 (0.047-24)			256 (8-1024)
**Cefuroxime**	**02/017**			12.7		*Staphylococcus epidermidis*			0.125		256
	**02/018**			44.2		*Cutibacterium acnes*			0.064		128
	**02/020**			11		*Micrococcus luteus*			0.38		16

Antimicrobial prophylaxis	Patient	Free plasma concentration (mg/L)				Bacterial species	MIC (mg/L)				Reciprocal SBT
		CFZ	GEN	CXM	VAN		CFZ	GEN	CXM	VAN	
	**02/021**			30.5		*Micrococcus luteus*			0.5		64
	**02/022**			20.5		*Staphylococcus hominis*			0.38		64
	**02/023**			28.0		*Cutibacterium acnes*			0.032		128
	**02/026**			38.5		*Micrococcus luteus*			0.5		128
	**02/027**			29.3		*Cutibacterium acnes*			0.016		1024
	**02/058**			23.2		*Staphylococcus epidermidis*			0.75		64
						Staphylococcus simulans			2		16
						Cutibacterium avidum			0.25		128
	**02/059**			24.3		*Kocuria rhizophila*			0.094		16
						Micrococcus luteus			0.38		16
	**02/060**			30.3		*Staphylococcus epidermidis*			0.25		128
						Staphylococcus hominis			1		32
						Staphylococcus capitis			0.75		8
						Cutibacterium acnes			0.064		128
	**02/062**			18.2		*Corynebacterium simulans*			0.75		128
						Corynebacterium mucifaciens			0.094		8
						Staphylococcus epidermidis [2]			16		16
						Staphylococcus hominis			0.5		32
						Micrococcus luteus			0.5		64
						Actinomyces neuii			0.023		128
	**02/024**			26.6		*Staphylococcus haemolyticus*			3		16
	**median (range)**			26.6 (11-44.2)					0.38 (0.016-16)		64 (8-1024)
**Cefuroxime +**	**01/051**		8.6	34.9		*Staphylococcus epidermidis*		0.25	0.38		64
						Staphylococcus hominis		0.38	0.5		256

Antimicrobial prophylaxis	Patient	Free plasma concentration (mg/L)				Bacterial species	MIC (mg/L)				Reciprocal SBT
		CFZ	GEN	CXM	VAN		CFZ	GEN	CXM	VAN	
Gentamicin	**01/063**		**11.9**	25.7		*Cutibacterium acnes*		**12**	0.032		64
	**01/066**		9.9	24.2		*Staphylococcus hominis*		0.064	0.75		8
	**01/070**		10.6	25.1		*Cutibacterium acnes*		8	0.047		256
						Staphylococcus saccharolyticus		0.25	0.023		256
	**02/029**		14.4	14.5		*Cutibacterium acnes*		8	0.5		32
						Staphylococcus epidermidis		0.25	0.19		256
	**02/030**		9.2	31.0		*Staphylococcus epidermidis*		0.064	0.38		128
	**02/035**		**9.9**	17.1		*Cutibacterium acnes*		**12**	0.023		ND
	**02/043**		**8.5**	21.8		*Cutibacterium acnes*		**12**	0.016		256
						Staphylococcus epidermidis		0.25	0.25		256
						Staphylococcus hominis		0.094	0.25		1024
	**02/032**		ND	ND		*Staphylococcus epidermidis*		ND	ND		ND
						Staphylococcus warneri		ND	ND		ND
	**median (range)**		9.9 (8.5-14.4)	24.7 (14.5-34.9)				0.25 (0.064-12)	0.25 (0.016-0.75)		256 (8-1024)
Vancomycin	**02/025**				5.2	*Staphylococcus epidermidis*				1.5	8
						Staphylococcus warneri				0.25	16
	**02/049**				7.5	*Staphylococcus epidermidis*				2	8
	**02/050**				19.0	*Micrococcus luteus*				0.25	256
						Cutibacterium acnes				0.125	64
	**02/052**				7.7	*Cutibacterium acnes*				0.25	32
	**02/054**				7.5	*Cutibacterium acnes*				0.25	64
	**02/015**				4.6	*Cutibacterium acnes*				0.25	32
	**median (range)**				7.6 (4.6-19.0)					0.25 (0.125-2)	32 (8-256)

Antimicrobial prophylaxis	Patient	Free plasma concentration (mg/L)				Bacterial species	MIC (mg/L)				Reciprocal SBT
		CFZ	GEN	CXM	VAN		CFZ	GEN	CXM	VAN	
Vancomycin + Gentamicin	**01/014**		**14.5**		8.5	*Cutibacterium acnes*		**24**		0.25	128
	**01/041**		15.2		8	*Cutibacterium acnes*		8		0.19	64
						Staphylococcus epidermidis		0.047		1.5	64
	**01/045**		13.3		8	*Dermabacter hominis*		8		0.25	128
						Staphylococcus epidermidis		0.047		1.5	256
	**01/052**		9.8		5.4	*Staphylococcus caprae*		0.25		0.75	256
	**01/053**		11.6		5.1	*Cutibacterium acnes*		8		0.25	16
	**01/046**		13.9		7.8	*Cutibacterium acnes*		8		0.19	32
	**median (range)**		13.6 (9.8-15.2)		7.9 (5.1-8.5)			8 (0.047-24)		0.25 (0.19-1.5)	96 (16-256)

CFZ, cefazolin; GEN, gentamicin CXM, cefuroxime; VAN, vancomycin; MIC, minimum inhibitory concentration; SBT, serum bactericidal titer; ND, not done. The cases in which the plasma concentrations were lower than the MIC are indicated in boldface.

[1]Patient 02/004 had two *S. epidermidis* isolates with different antibiotypes.

[2]Resistance to oxacillin.
